# *catena*-Poly[[diaqua­cadmium(II)]bis­(μ-2,2-dimethyl­butane­dioato-κ^4^ *O*,*O*′:*O*′′,*O*′′′)[diaqua­cadmium(II)]-μ-1,4-bis­(3-pyridylmeth­yl)piperazine-κ^2^ *N* ^3^:*N* ^3′^]

**DOI:** 10.1107/S1600536810018799

**Published:** 2010-05-26

**Authors:** Amy L. Pochodylo, Robert L. LaDuca

**Affiliations:** a Lyman Briggs College, Department of Chemistry, Michigan State University, East Lansing, MI 48825, USA

## Abstract

In the title compound, [Cd_2_(C_6_H_8_O_4_)_2_(C_16_H_20_N_4_)(H_2_O)_4_]_*n*_, penta­gonal-bipyramidally coordinated Cd^II^ ions are connected into {Cd_2_(2,2-dimethyl­succinate)_2_(H_2_O)_4_} centrosymmetric dimeric clusters. In turn, these clusters are linked by tethering 1,4-bis­(3-pyridylmeth­yl)piperazine (3-bpmp) ligands into [Cd_2_(2,2-dimethyl­succinate)_2_(3-bpmp)(H_2_O)_4_]_n_ coordination polymer chains. The chain motifs are oriented parallel to [1
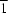
0]. Individual chains are connected into supra­molecular layers *via* O—H⋯N and O—H⋯O hydrogen-bonding mechanisms.

## Related literature

For other dicarboxyl­ate coordination polymers containing 3-bpmp ligands, see: Johnston *et al.* (2008 ▶). For the preparation of 3-bpmp, see: Niu *et al.* (2001 ▶).
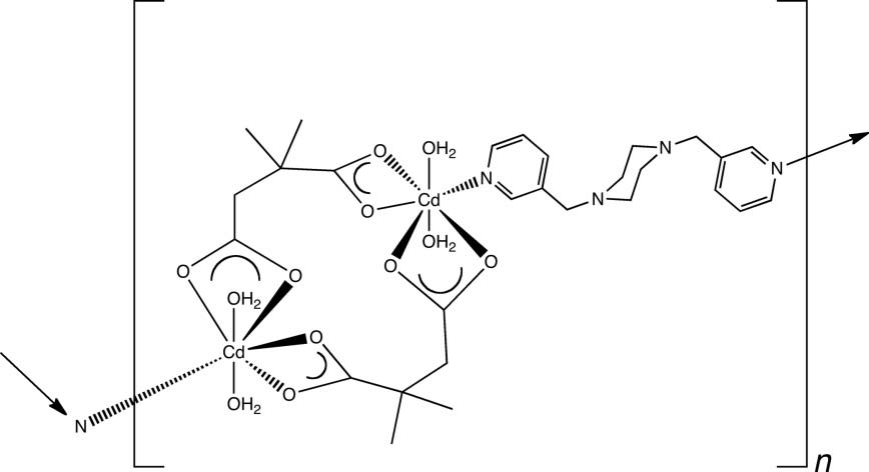


## Experimental

### 

#### Crystal data

[Cd_2_(C_6_H_8_O_4_)_2_(C_16_H_20_N_4_)(H_2_O)_4_]
*M* *_r_* = 426.74
Triclinic, 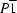

*a* = 9.275 (3) Å
*b* = 10.378 (3) Å
*c* = 10.625 (5) Å
α = 114.461 (3)°
β = 101.274 (3)°
γ = 106.687 (2)°
*V* = 831.6 (5) Å^3^
*Z* = 2
Mo *K*α radiation
μ = 1.34 mm^−1^
*T* = 173 K
0.26 × 0.18 × 0.13 mm

#### Data collection

Bruker APEXII diffractometer
Absorption correction: multi-scan (*SADABS*; Sheldrick, 1996 ▶) *T*_min_ = 0.723, *T*_max_ = 0.840
12027 measured reflections
3047 independent reflections
2909 reflections with *I* > 2σ(*I*)
*R*_int_ = 0.058

#### Refinement

*R*[*F* ^2^ > 2σ(*F* ^2^)] = 0.035
*wR*(*F* ^2^) = 0.099
*S* = 1.16
3047 reflections
222 parameters
6 restraints
H atoms treated by a mixture of independent and constrained refinement
Δρ_max_ = 0.65 e Å^−3^
Δρ_min_ = −1.07 e Å^−3^

### 

Data collection: *APEX2* (Bruker, 2006 ▶); cell refinement: *SAINT* (Bruker, 2006 ▶); data reduction: *SAINT*; program(s) used to solve structure: *SHELXS97* (Sheldrick, 2008 ▶); program(s) used to refine structure: *SHELXL97* (Sheldrick, 2008 ▶); molecular graphics: *CrystalMaker* (Palmer, 2007 ▶); software used to prepare material for publication: *SHELXL97*.

## Supplementary Material

Crystal structure: contains datablocks I, global. DOI: 10.1107/S1600536810018799/lh5049sup1.cif

Structure factors: contains datablocks I. DOI: 10.1107/S1600536810018799/lh5049Isup2.hkl

Additional supplementary materials:  crystallographic information; 3D view; checkCIF report

## Figures and Tables

**Table 1:** Hydrogen-bond geometry (Å, °)

*D*—H⋯*A*	*D*—H	H⋯*A*	*D*⋯*A*	*D*—H⋯*A*
O5—H5*C*⋯O4	0.84 (4)	1.93 (2)	2.705 (3)	154 (4)
O5—H5*D*⋯O1^i^	0.83 (2)	1.99 (2)	2.744 (3)	152 (3)
O6—H6*C*⋯O3^ii^	0.85 (2)	1.84 (2)	2.679 (3)	171 (4)
O6—H6*D*⋯N2^iii^	0.83 (2)	2.03 (2)	2.851 (4)	173 (4)

Symmetry codes: (i) 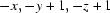; (ii) 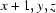; (iii) 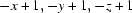.

**Fig. 1.**
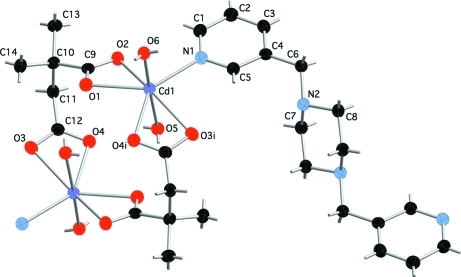
The coordination environment and dinuclear cluster of the title compound, showing 50% probability ellipsoids and partial atom numbering scheme. Hydrogen atom positions are shown as grey sticks. Color codes: violet Cd, red O, light blue N, black C. Symmetry code: (i) -x, -y + 1, -z + 1.

**Fig. 2.**
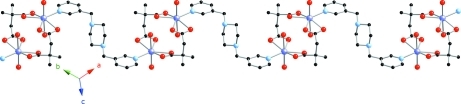
A single [Cd2(2,2-dimethylsuccinate)2(H2O)4 (3-bpmp)]n coordination polymer chain

**Fig. 3.**
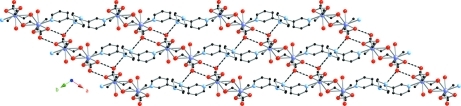
Supramolecular layer of [Cd2(2,2-dimethylsuccinate)2(H2O)4 (3-bpmp)]n chains. Although the H atoms have been omitted the O—H···N and O—H···O hydrogen bonds are shown as dashed lines between the donor and acceptor atoms.

**Fig. 4.**
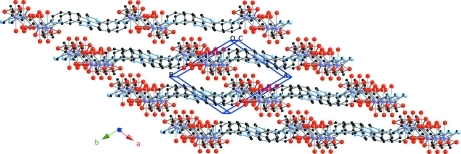
Stacking of supramolecular layers in the title compound.

## supplementary crystallographic information

### Comment

Recently we have been investigating bis(3-pyridylmethyl)piperazine (3-bpmp) as a neutral dipodal tethering ligand for the construction of divalent metal coordination polymers in tandem with aromatic dicarboxylate ligands (Johnston *et al.*,  2008). This chemistry has been extended into an aliphatic dicarboxylate system with the synthesis of the title compound.

The asymmetric unit of the title compound contains a Cd^II^ ion, one 2,2-dimethylsuccinate ligand, two aqua ligands, and one-half of a 3-bpmp ligand whose central piperazinyl ring is situated over a crystallographic inversion centre. The Cd^II^ ion is pentagonal bipyramidally coordinated in a {CdO_6_N} environment, with its apical positions occupied by aqua ligands. Its basal plane consists of two chelating carboxylate groups from two 2,2-dimethylsuccinate ligands and one pyridyl N donor atom from a 3-bpmp ligand. A pair of Cd^II^ ions is aggregated into a centrosymmetric {Cd_2_(H_2_O)_4_(2,2-dimethylsuccinate)_2_} dinuclear cluster (Fig. 1) by two bis(chelating) 2,2-dimethylsuccinate ligands, which adopt a *gauche* conformation.

{Cd_2_(H_2_O)_4_(2,2-dimethylsuccinate)_2_} dinuclear clusters are connected by tethering 3-bpmp ligands into one-dimensional [Cd_2_(2,2-dimethylsuccinate)_2_(H_2_O)_4_(3-bpmp)]_n_ coordination polymer chains, which are oriented parallel to the (1 1 0) crystal direction (Fig. 2). The through-ligand Cd···Cd contact distance is 13.381 (6) Å. Individual chains are connected into supramolecular *pseudo* layers (Fig. 3) *via* O—H···N and O—H···O interactions (Table 1). Within the *pseudo* layers, aqua ligands (O6) donate hydrogen bonds to piperazinyl N atoms of 3-bpmp ligands and ligated 2,2-dimethylsuccinate carboxylate O atoms in neighboring chains. Neighboring *pseudo* layers stack into the three-dimensional crystal structure (Fig. 4) of the title compound by crystal packing forces.

### Experimental

All starting materials were obtained commercially, except for 3-bpmp, which was prepared by a published procedure (Niu *et al.*, 2001). A mixture of cadmium nitrate tetrahydrate (114 mg, 0.37 mmol), 2,2-dimethylsuccinic acid (54 mg, 0.37 mmol), 3-bpmp (199 mg, 0.742 mmol) and 10.0 g water (550 mmol) was placed into a 23 ml Teflon-lined Parr acid digestion bomb, which was then heated under autogenous pressure at 393 K for 72 h. Colourless blocks of the title compound (57 mg, 26% yield) were isolated after washing with distilled water and acetone, and drying in air.

### Refinement

All H atoms bound to C atoms were placed in calculated positions, with C—H = 0.95 Å, and refined in riding mode with *U*_iso_ = 1.2*U*_eq_(C). The H atoms bound to the aqua ligand O atom were found in a difference Fourier map, restrained with with O—H = 0.85 Å and refined with *U*_iso_ = 1.2*U*_eq_(O).

### Crystal data

**Table:**

[Cd_2_(C_6_H_8_O_4_)_2_(C_16_H_20_N_4_)(H_2_O)_4_]	*Z* = 2
*M**_r_* = 426.74	*F*(000) = 432
Triclinic, *P*1	*D*_x_ = 1.704 Mg m^−^^3^
Hall symbol: -P 1	Mo *K*α radiation, λ = 0.71073 Å
*a* = 9.275 (3) Å	Cell parameters from 12027 reflections
*b* = 10.378 (3) Å	θ = 2.3–25.4°
*c* = 10.625 (5) Å	µ = 1.34 mm^−^^1^
α = 114.461 (3)°	*T* = 173 K
β = 101.274 (3)°	Block, colourless
γ = 106.687 (2)°	0.26 × 0.18 × 0.13 mm
*V* = 831.6 (5) Å^3^	

### Data collection

**Table:**

Bruker APEXII diffractometer	3047 independent reflections
Radiation source: fine-focus sealed tube	2909 reflections with *I* > 2σ(*I*)
graphite	*R*_int_ = 0.058
ω–φ scans	θ_max_ = 25.4°, θ_min_ = 2.3°
Absorption correction: multi-scan (*SADABS*; Sheldrick, 1996)	*h* = −11→11
*T*_min_ = 0.723, *T*_max_ = 0.840	*k* = −12→12
12027 measured reflections	*l* = −12→12

### Refinement

**Table:**

Refinement on *F*^2^	Primary atom site location: structure-invariant direct methods
Least-squares matrix: full	Secondary atom site location: difference Fourier map
*R*[*F*^2^ > 2σ(*F*^2^)] = 0.035	Hydrogen site location: inferred from neighbouring sites
*wR*(*F*^2^) = 0.099	H atoms treated by a mixture of independent and constrained refinement
*S* = 1.16	*w* = 1/[σ^2^(*F*_o_^2^) + (0.0539*P*)^2^ + 0.0373*P*] where *P* = (*F*_o_^2^ + 2*F*_c_^2^)/3
3047 reflections	(Δ/σ)_max_ < 0.001
222 parameters	Δρ_max_ = 0.65 e Å^−^^3^
6 restraints	Δρ_min_ = −1.06 e Å^−^^3^

### Special details

**Table:**

Experimental. The largest peak of 0.645 e^-^ Å^3^ was located 0.90 Å from Cd1. The largest hole of -1.065 e^-^ Å^3^ was located 0.94 Å from Cd1.
Geometry. All esds (except the esd in the dihedral angle between two l.s. planes) are estimated using the full covariance matrix. The cell esds are taken into account individually in the estimation of esds in distances, angles and torsion angles; correlations between esds in cell parameters are only used when they are defined by crystal symmetry. An approximate (isotropic) treatment of cell esds is used for estimating esds involving l.s. planes.
Refinement. Refinement of *F*^2^ against ALL reflections. The weighted *R*-factor wR and goodness of fit *S* are based on *F*^2^, conventional *R*-factors *R* are based on *F*, with *F* set to zero for negative *F*^2^. The threshold expression of *F*^2^ > σ(*F*^2^) is used only for calculating *R*-factors(gt) etc. and is not relevant to the choice of reflections for refinement. *R*-factors based on *F*^2^ are statistically about twice as large as those based on *F*, and *R*- factors based on ALL data will be even larger.

### Fractional atomic coordinates and isotropic or equivalent isotropic displacement parameters (Å^2^)

**Table:**

	*x*	*y*	*z*	*U*_iso_*/*U*_eq_	
Cd1	0.18482 (2)	0.45331 (2)	0.36199 (2)	0.03562 (13)	
O1	0.0456 (2)	0.6107 (3)	0.3511 (2)	0.0425 (5)	
O2	−0.0444 (3)	0.3806 (3)	0.1510 (3)	0.0436 (5)	
O3	−0.3418 (3)	0.5696 (3)	0.4297 (3)	0.0452 (5)	
O4	−0.2497 (3)	0.3895 (3)	0.3798 (3)	0.0423 (5)	
O5	−0.0262 (3)	0.2695 (3)	0.3722 (3)	0.0413 (5)	
H5C	−0.098 (4)	0.297 (5)	0.346 (4)	0.050*	
H5D	−0.007 (4)	0.295 (4)	0.461 (2)	0.050*	
O6	0.4016 (3)	0.6335 (3)	0.3687 (3)	0.0389 (5)	
H6C	0.483 (3)	0.611 (4)	0.379 (4)	0.047*	
H6D	0.430 (4)	0.720 (3)	0.441 (3)	0.047*	
N1	0.2421 (3)	0.2552 (3)	0.2065 (3)	0.0369 (5)	
N2	0.5008 (3)	0.0578 (3)	0.3970 (3)	0.0367 (5)	
C1	0.1678 (4)	0.1796 (4)	0.0587 (4)	0.0418 (7)	
H1	0.0989	0.2150	0.0165	0.050*	
C2	0.1890 (4)	0.0519 (4)	−0.0333 (4)	0.0460 (8)	
H2	0.1354	0.0005	−0.1376	0.055*	
C3	0.2875 (4)	−0.0005 (4)	0.0262 (4)	0.0435 (7)	
H3	0.2997	−0.0904	−0.0364	0.052*	
C4	0.3700 (4)	0.0788 (4)	0.1793 (4)	0.0375 (6)	
C5	0.3413 (4)	0.2057 (4)	0.2633 (4)	0.0392 (7)	
H5	0.3956	0.2609	0.3677	0.047*	
C6	0.4891 (4)	0.0317 (4)	0.2474 (4)	0.0405 (7)	
H6A	0.4588	−0.0801	0.1810	0.049*	
H6B	0.5971	0.0900	0.2520	0.049*	
C7	0.3498 (4)	−0.0430 (4)	0.3945 (4)	0.0402 (7)	
H7A	0.3231	−0.1530	0.3243	0.048*	
H7B	0.2605	−0.0176	0.3595	0.048*	
C8	0.6337 (4)	0.0216 (4)	0.4535 (4)	0.0400 (7)	
H8A	0.7361	0.0906	0.4580	0.048*	
H8B	0.6126	−0.0871	0.3846	0.048*	
C9	−0.0570 (4)	0.5055 (4)	0.2235 (3)	0.0373 (7)	
C10	−0.2056 (4)	0.5280 (4)	0.1593 (4)	0.0390 (7)	
C11	−0.3507 (4)	0.4289 (4)	0.1793 (3)	0.0400 (7)	
H11A	−0.4454	0.4496	0.1483	0.048*	
H11B	−0.3787	0.3173	0.1142	0.048*	
C12	−0.3141 (3)	0.4641 (4)	0.3390 (3)	0.0377 (7)	
C13	−0.2436 (4)	0.4650 (4)	−0.0073 (4)	0.0471 (8)	
H13A	−0.3426	0.4721	−0.0503	0.071*	
H13B	−0.2585	0.3561	−0.0552	0.071*	
H13C	−0.1540	0.5264	−0.0228	0.071*	
C14	−0.1754 (4)	0.6995 (4)	0.2367 (4)	0.0478 (8)	
H14A	−0.0781	0.7601	0.2292	0.072*	
H14B	−0.1607	0.7363	0.3414	0.072*	
H14C	−0.2681	0.7121	0.1892	0.072*	

### Atomic displacement parameters (Å^2^)

**Table:**

	*U*^11^	*U*^22^	*U*^33^	*U*^12^	*U*^13^	*U*^23^
Cd1	0.03706 (18)	0.03714 (18)	0.03548 (19)	0.01797 (13)	0.01430 (13)	0.01828 (14)
O1	0.0399 (12)	0.0476 (12)	0.0378 (12)	0.0189 (10)	0.0107 (10)	0.0205 (10)
O2	0.0480 (13)	0.0440 (12)	0.0446 (12)	0.0250 (10)	0.0183 (10)	0.0223 (11)
O3	0.0475 (13)	0.0516 (14)	0.0391 (12)	0.0263 (11)	0.0185 (10)	0.0197 (11)
O4	0.0431 (12)	0.0469 (12)	0.0427 (12)	0.0227 (10)	0.0174 (10)	0.0239 (11)
O5	0.0441 (13)	0.0429 (13)	0.0388 (13)	0.0205 (11)	0.0149 (11)	0.0205 (11)
O6	0.0414 (12)	0.0383 (12)	0.0373 (13)	0.0188 (10)	0.0139 (10)	0.0177 (11)
N1	0.0372 (13)	0.0380 (13)	0.0382 (14)	0.0173 (11)	0.0150 (11)	0.0194 (12)
N2	0.0397 (13)	0.0369 (13)	0.0395 (14)	0.0181 (11)	0.0170 (11)	0.0215 (12)
C1	0.0429 (17)	0.0438 (17)	0.0412 (17)	0.0193 (14)	0.0141 (14)	0.0230 (15)
C2	0.0541 (19)	0.0447 (18)	0.0343 (17)	0.0216 (15)	0.0125 (15)	0.0161 (15)
C3	0.0505 (18)	0.0384 (16)	0.0423 (18)	0.0214 (14)	0.0194 (15)	0.0175 (15)
C4	0.0427 (16)	0.0366 (15)	0.0379 (16)	0.0167 (13)	0.0190 (14)	0.0204 (13)
C5	0.0393 (16)	0.0405 (16)	0.0379 (16)	0.0172 (13)	0.0135 (13)	0.0193 (14)
C6	0.0492 (18)	0.0394 (16)	0.0417 (17)	0.0224 (14)	0.0221 (14)	0.0226 (15)
C7	0.0386 (15)	0.0405 (16)	0.0454 (17)	0.0177 (13)	0.0165 (13)	0.0234 (15)
C8	0.0404 (16)	0.0403 (16)	0.0456 (17)	0.0197 (13)	0.0181 (13)	0.0236 (14)
C9	0.0398 (16)	0.0461 (17)	0.0380 (16)	0.0211 (14)	0.0201 (14)	0.0264 (15)
C10	0.0426 (17)	0.0429 (17)	0.0370 (17)	0.0206 (14)	0.0142 (14)	0.0227 (15)
C11	0.0368 (16)	0.0447 (17)	0.0396 (17)	0.0207 (14)	0.0114 (13)	0.0205 (15)
C12	0.0316 (16)	0.0445 (18)	0.0385 (16)	0.0153 (14)	0.0144 (13)	0.0217 (15)
C13	0.053 (2)	0.058 (2)	0.0372 (17)	0.0269 (17)	0.0162 (15)	0.0268 (16)
C14	0.058 (2)	0.0441 (18)	0.052 (2)	0.0275 (16)	0.0221 (17)	0.0280 (16)

### Geometric parameters (Å, °)

**Table:**

Cd1—O6	2.284 (2)	C3—H3	0.9500
Cd1—N1	2.323 (3)	C4—C5	1.386 (4)
Cd1—O5	2.364 (2)	C4—C6	1.507 (5)
Cd1—O4^i^	2.374 (3)	C5—H5	0.9500
Cd1—O1	2.378 (2)	C6—H6A	0.9900
Cd1—O2	2.435 (2)	C6—H6B	0.9900
Cd1—O3^i^	2.540 (2)	C7—C8^ii^	1.506 (4)
O1—C9	1.268 (4)	C7—H7A	0.9900
O2—C9	1.258 (4)	C7—H7B	0.9900
O3—C12	1.256 (4)	C8—C7^ii^	1.506 (4)
O4—C12	1.259 (4)	C8—H8A	0.9900
O5—H5C	0.84 (4)	C8—H8B	0.9900
O5—H5D	0.830 (18)	C9—C10	1.539 (4)
O6—H6C	0.851 (18)	C10—C14	1.528 (4)
O6—H6D	0.825 (18)	C10—C13	1.532 (4)
N1—C5	1.336 (4)	C10—C11	1.553 (5)
N1—C1	1.342 (4)	C11—C12	1.523 (4)
N2—C6	1.474 (4)	C11—H11A	0.9900
N2—C7	1.475 (4)	C11—H11B	0.9900
N2—C8	1.478 (4)	C13—H13A	0.9800
C1—C2	1.379 (5)	C13—H13B	0.9800
C1—H1	0.9500	C13—H13C	0.9800
C2—C3	1.369 (5)	C14—H14A	0.9800
C2—H2	0.9500	C14—H14B	0.9800
C3—C4	1.397 (5)	C14—H14C	0.9800
			
H14B—C14—H14C	109.5	C5—C4—C6	122.3 (3)
O6—Cd1—N1	90.10 (9)	C3—C4—C6	121.2 (3)
O6—Cd1—O5	175.51 (7)	N1—C5—C4	124.1 (3)
N1—Cd1—O5	90.19 (9)	N1—C5—H5	118.0
O6—Cd1—O4^i^	90.71 (8)	C4—C5—H5	118.0
N1—Cd1—O4^i^	138.03 (8)	N2—C6—C4	115.3 (3)
O5—Cd1—O4^i^	86.08 (8)	N2—C6—H6A	108.5
O6—Cd1—O1	86.80 (8)	C4—C6—H6A	108.5
N1—Cd1—O1	140.28 (8)	N2—C6—H6B	108.5
O5—Cd1—O1	95.83 (8)	C4—C6—H6B	108.5
O4^i^—Cd1—O1	81.63 (8)	H6A—C6—H6B	107.5
O6—Cd1—O2	106.73 (8)	N2—C7—C8^ii^	110.9 (3)
N1—Cd1—O2	88.92 (8)	N2—C7—H7A	109.5
O5—Cd1—O2	77.76 (8)	C8^ii^—C7—H7A	109.5
O4^i^—Cd1—O2	130.56 (8)	N2—C7—H7B	109.5
O1—Cd1—O2	54.59 (7)	C8^ii^—C7—H7B	109.5
O6—Cd1—O3^i^	95.93 (8)	H7A—C7—H7B	108.1
N1—Cd1—O3^i^	85.50 (8)	N2—C8—C7^ii^	111.1 (2)
O5—Cd1—O3^i^	79.62 (8)	N2—C8—H8A	109.4
O4^i^—Cd1—O3^i^	52.70 (8)	C7^ii^—C8—H8A	109.4
O1—Cd1—O3^i^	134.21 (7)	N2—C8—H8B	109.4
O2—Cd1—O3^i^	156.67 (9)	C7^ii^—C8—H8B	109.4
O6—Cd1—C9	98.97 (9)	H8A—C8—H8B	108.0
N1—Cd1—C9	115.36 (9)	O2—C9—O1	121.9 (3)
O5—Cd1—C9	84.95 (9)	O2—C9—C10	119.1 (3)
O4^i^—Cd1—C9	105.92 (9)	O1—C9—C10	118.9 (3)
O1—Cd1—C9	27.43 (8)	O2—C9—Cd1	62.34 (16)
O2—Cd1—C9	27.24 (8)	O1—C9—Cd1	59.80 (16)
O3^i^—Cd1—C9	154.14 (8)	C10—C9—Cd1	171.7 (2)
C9—O1—Cd1	92.76 (18)	C14—C10—C13	110.1 (3)
C9—O2—Cd1	90.42 (18)	C14—C10—C9	110.8 (3)
C12—O3—Cd1^i^	89.11 (19)	C13—C10—C9	109.0 (3)
C12—O4—Cd1^i^	96.79 (19)	C14—C10—C11	111.3 (3)
Cd1—O5—H5C	96 (3)	C13—C10—C11	107.9 (3)
Cd1—O5—H5D	107 (3)	C9—C10—C11	107.5 (3)
H5C—O5—H5D	108 (3)	C12—C11—C10	112.2 (3)
Cd1—O6—H6C	111 (3)	C12—C11—H11A	109.2
Cd1—O6—H6D	110 (3)	C10—C11—H11A	109.2
H6C—O6—H6D	106 (3)	C12—C11—H11B	109.2
C5—N1—C1	118.2 (3)	C10—C11—H11B	109.2
C5—N1—Cd1	120.3 (2)	H11A—C11—H11B	107.9
C1—N1—Cd1	121.3 (2)	O3—C12—O4	120.7 (3)
C6—N2—C7	111.3 (3)	O3—C12—C11	120.6 (3)
C6—N2—C8	108.4 (2)	O4—C12—C11	118.6 (3)
C7—N2—C8	108.8 (2)	C10—C13—H13A	109.5
N1—C1—C2	121.6 (3)	C10—C13—H13B	109.5
N1—C1—H1	119.2	H13A—C13—H13B	109.5
C2—C1—H1	119.2	C10—C13—H13C	109.5
C3—C2—C1	119.8 (3)	H13A—C13—H13C	109.5
C3—C2—H2	120.1	H13B—C13—H13C	109.5
C1—C2—H2	120.1	C10—C14—H14A	109.5
C2—C3—C4	119.8 (3)	C10—C14—H14B	109.5
C2—C3—H3	120.1	H14A—C14—H14B	109.5
C4—C3—H3	120.1	C10—C14—H14C	109.5
C5—C4—C3	116.5 (3)	H14A—C14—H14C	109.5
			
O6—Cd1—O1—C9	116.54 (18)	C5—C4—C6—N2	34.4 (4)
N1—Cd1—O1—C9	30.2 (2)	C3—C4—C6—N2	−148.3 (3)
O5—Cd1—O1—C9	−67.12 (18)	C6—N2—C7—C8^ii^	176.9 (2)
O4^i^—Cd1—O1—C9	−152.27 (18)	C8—N2—C7—C8^ii^	57.5 (3)
O2—Cd1—O1—C9	3.36 (16)	C6—N2—C8—C7^ii^	−178.9 (2)
O3^i^—Cd1—O1—C9	−148.30 (17)	C7—N2—C8—C7^ii^	−57.7 (4)
O6—Cd1—O2—C9	−76.80 (18)	Cd1—O2—C9—O1	6.1 (3)
N1—Cd1—O2—C9	−166.58 (17)	Cd1—O2—C9—C10	−170.7 (2)
O5—Cd1—O2—C9	102.99 (18)	Cd1—O1—C9—O2	−6.2 (3)
O4^i^—Cd1—O2—C9	29.1 (2)	Cd1—O1—C9—C10	170.6 (2)
O1—Cd1—O2—C9	−3.38 (16)	O6—Cd1—C9—O2	109.28 (17)
O3^i^—Cd1—O2—C9	117.4 (2)	N1—Cd1—C9—O2	14.88 (19)
O6—Cd1—N1—C5	89.2 (2)	O5—Cd1—C9—O2	−72.93 (17)
O5—Cd1—N1—C5	−86.3 (2)	O4^i^—Cd1—C9—O2	−157.39 (16)
O4^i^—Cd1—N1—C5	−1.9 (3)	O1—Cd1—C9—O2	174.0 (3)
O1—Cd1—N1—C5	174.34 (19)	O3^i^—Cd1—C9—O2	−126.3 (2)
O2—Cd1—N1—C5	−164.0 (2)	O6—Cd1—C9—O1	−64.73 (18)
O3^i^—Cd1—N1—C5	−6.7 (2)	N1—Cd1—C9—O1	−159.13 (16)
C9—Cd1—N1—C5	−170.8 (2)	O5—Cd1—C9—O1	113.06 (18)
O6—Cd1—N1—C1	−96.0 (2)	O4^i^—Cd1—C9—O1	28.61 (18)
O5—Cd1—N1—C1	88.5 (2)	O2—Cd1—C9—O1	−174.0 (3)
O4^i^—Cd1—N1—C1	172.8 (2)	O3^i^—Cd1—C9—O1	59.7 (3)
O1—Cd1—N1—C1	−10.9 (3)	O2—C9—C10—C14	−162.7 (3)
O2—Cd1—N1—C1	10.7 (2)	O1—C9—C10—C14	20.4 (4)
O3^i^—Cd1—N1—C1	168.1 (2)	O2—C9—C10—C13	−41.4 (4)
C9—Cd1—N1—C1	4.0 (3)	O1—C9—C10—C13	141.7 (3)
C5—N1—C1—C2	1.5 (5)	O2—C9—C10—C11	75.4 (3)
Cd1—N1—C1—C2	−173.4 (2)	O1—C9—C10—C11	−101.5 (3)
N1—C1—C2—C3	0.2 (5)	C14—C10—C11—C12	−68.5 (3)
C1—C2—C3—C4	−2.1 (5)	C13—C10—C11—C12	170.5 (3)
C2—C3—C4—C5	2.1 (5)	C9—C10—C11—C12	53.1 (3)
C2—C3—C4—C6	−175.2 (3)	Cd1^i^—O3—C12—O4	8.2 (3)
C1—N1—C5—C4	−1.4 (5)	Cd1^i^—O3—C12—C11	−169.0 (2)
Cd1—N1—C5—C4	173.5 (2)	Cd1^i^—O4—C12—O3	−8.9 (3)
C3—C4—C5—N1	−0.4 (5)	Cd1^i^—O4—C12—C11	168.4 (2)
C6—C4—C5—N1	176.9 (3)	C10—C11—C12—O3	89.1 (3)
C7—N2—C6—C4	66.3 (3)	C10—C11—C12—O4	−88.2 (3)
C8—N2—C6—C4	−174.1 (3)		

Symmetry codes: (i) −*x*, −*y*+1, −*z*+1; (ii) −*x*+1, −*y*, −*z*+1.

### Hydrogen-bond geometry (Å, °)

**Table:**

*D*—H···*A*	*D*—H	H···*A*	*D*···*A*	*D*—H···*A*
O5—H5C···O4	0.84 (4)	1.93 (2)	2.705 (3)	154 (4)
O5—H5D···O1^i^	0.83 (2)	1.99 (2)	2.744 (3)	152 (3)
O6—H6C···O3^iii^	0.85 (2)	1.84 (2)	2.679 (3)	171 (4)
O6—H6D···N2^iv^	0.83 (2)	2.03 (2)	2.851 (4)	173 (4)

Symmetry codes: (i) −*x*, −*y*+1, −*z*+1; (iii) *x*+1, *y*, *z*; (iv) −*x*+1, −*y*+1, −*z*+1.
